# Association of the non-high-density lipoprotein cholesterol to high-density lipoprotein cholesterol ratio (NHHR) with COPD prevalence and all-cause mortality: a population-based study based on NHANES 2007–2016

**DOI:** 10.3389/fmed.2025.1533744

**Published:** 2025-04-03

**Authors:** Yu Liu, Zheng Fan, Hongmei Ren, Cuixia Zheng

**Affiliations:** Department of Respiratory and Critical Care Medicine, Yangpu Hospital, School of Medicine, Tongji University, Shanghai, China

**Keywords:** COPD prevalence, all-cause mortality, NHHR, NHANES, lipid

## Abstract

**Background:**

The non-high-density lipoprotein cholesterol to high-density lipoprotein cholesterol ratio (NHHR) plays a potential role in metabolic and cardiovascular diseases. However, its association with chronic obstructive pulmonary disease (COPD) is not well-defined. Here, we aim to investigate the potential association of NHHR with both the prevalence of COPD and all-cause mortality among individuals with COPD.

**Methods:**

This population-based NHANES (2007–2016) study utilized weighted statistical analyses. Multivariable logistic regression assessed the NHHR-COPD prevalence association, with restricted cubic spline (RCS) testing for non-linearity. The association between NHHR and all-cause mortality in COPD was evaluated using Cox proportional hazards models and Kaplan-Meier, with RCS testing for non-linearity. Subgroup and sensitivity analyses confirmed the findings’ reliability.

**Results:**

This study included 6349 participants, of whom 1271 were diagnosed with COPD. Participants in the highest NHHR tertile demonstrated 62% higher odds of COPD prevalence compared to those in the lowest tertile (OR = 1.62, 95% CI:1.11–2.39, *P* = 0.017). Results from RCS analysis indicated a nonlinear relationship between NHHR and the prevalence of COPD (*P* for nonlinear = 0.007), with the curve demonstrating an inverted L-shape. Over an average follow-up period of 93 months, 320 participants with COPD died. In the weighted Kaplan-Meier survival analysis, participants with COPD in the lower NHHR tertile demonstrated greater cumulative probability of all-cause mortality compared to higher tertiles (*P* < 0.001). Weighted multivariable Cox regression models revealed an inverse association between NHHR levels and COPD all-cause mortality, with the highest NHHR tertile showing 11% lower likelihood of COPD all-cause mortality relative to the lowest tertile (HR = 0.89, 95% CI:0.80–0.99, *P* = 0.027). In addition, RCS analysis demonstrated a significant negative linear association between NHHR levels and all-cause mortality in COPD patients (*P* for nonlinear = 0.081). Subgroup and sensitivity analyses further confirmed the associations of NHHR on both morbidity and all-cause mortality.

**Conclusion:**

Higher NHHR levels were associated with increased COPD prevalence yet inversely correlated with all-cause mortality in COPD patients. These paradoxical associations underscore the need for COPD-specific lipid management strategies that balance disease progression and mortality risks.

## Introduction

Chronic obstructive pulmonary disease is a prevalent and debilitating respiratory condition characterized by airflow limitation that is not fully reversible. It encompasses chronic bronchitis and emphysema and is primarily caused by exposure to harmful particles or gases, most commonly from tobacco smoking (1, 2). COPD is the fourth leading cause of death worldwide, causing 3.5 million deaths in 2021, approximately 5% of all global deaths (3–5). The disease is associated with a high economic burden due to healthcare costs, hospitalizations, and loss of productivity (6). Accordingly, it is of utmost importance to identify modifiable risk factors of COPD to guide effective prevention and interventions.

COPD is known to be a systemic disease affecting not only the lungs but also various other organs and systems in the body. Common comorbidities of COPD include cardiovascular diseases, osteoporosis, and muscle wasting, which can further exacerbate the burden of the disease and significantly impact patients’ quality of life (7, 8). Most of these comorbidities belong to metabolic diseases and are closely related to dyslipidemia (9–11). Dyslipidemia is defined as an imbalance of plasma lipids and/or lipoproteins, such as triglycerides (TG), high-density lipoprotein cholesterol (HDL-C), and low-density lipoprotein cholesterol (LDL-C) (12). Interestingly, disturbances in lipid metabolism have also been observed in patients with COPD, with alterations in cholesterol levels and lipid profiles (13–16).

The non-high-density lipoprotein cholesterol (non-HDL-C) to HDL-C ratio (NHHR), a novel lipid composite indicator, reflects the balance between HDL-C and non-HDL-C, which are lipoproteins with different functions. As a biomarker, NHHR has been studied in cardiovascular diseases and diabetes and has shown the potential to outperform traditional lipid indicators (17–19). A study revealed that NHHR was superior to traditional lipid markers in predicting no-reflow development in patients with ST-elevated myocardial infarction (STEMI) (20). Additionally, there is a negative correlation between NHHR and glomerular filtration rate in chronic kidney disease (21) and a positive correlation with alanine aminotransferase/aspartate aminotransferase levels in non-alcoholic fatty liver disease (22, 23). These studies indicate that NHHR may be a valuable tool for predicting diseases related to metabolism.

Recently, there has been an increased focus on studying COPD and lipid metabolism (9, 24). Several studies have investigated the relationship between COPD and lipid profiles, with conflicting results. Some studies have shown decreased serum HDL levels or increased serum TG levels in patients with COPD (25, 26), while others have not found any significant changes in lipid profiles (27). Therefore, the relationship between NHHR and COPD needs to be further confirmed, especially the effect of NHHR on COPD all-cause mortality.

This study aims to investigate the association between NHHR and the prevalence and all-cause mortality of COPD using data from the National Health and Nutrition Examination Survey (NHANES), a nationally representative database of the United States population. By examining the relationship between NHHR and COPD outcomes, this research seeks to contribute to the understanding of the metabolic implications of COPD.

## Material and methods

### Data source and study population

National Health and Nutrition Examination Survey (NHANES) is a national survey conducted by the National Center for Health Statistics (NCHS) under the Centers for Disease Control and Prevention (CDC) to collect population health data. It has been conducted every 2 years since 1999. For this analysis, we used publicly available NHANES data from five cycles, conducted from 2007 to 2008 and 2015 to 2016. Each NHANES survey cycle was rigorously evaluated and approved by the NCHS Research Ethics Review Board, and all participants provided written informed consent.

**FIGURE 1:**
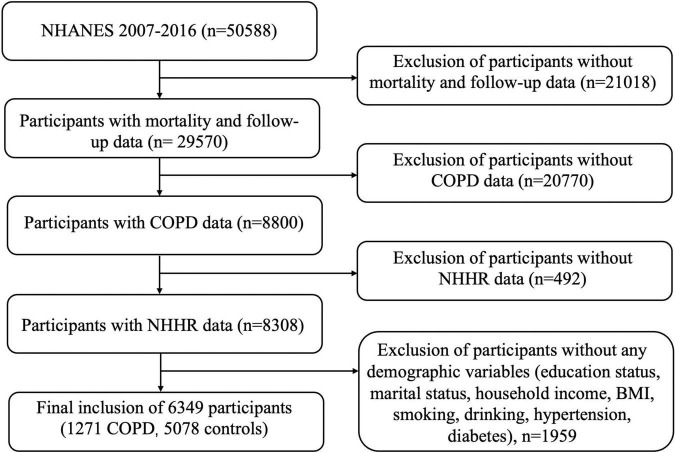
Flowchart of participant selection for this study.

We extracted and aggregated data on basic characteristics, socioeconomic characteristics information, lifestyle information, laboratory examination, and health questionnaires. Initially, a total of 50, 588 participants were included. Exclusions were made for the following individuals: (1) participants without mortality and follow-up data (*n* = 21018); (2) participants without COPD data (*n* = 20770); (3) missing NHHR data (*n* = 492); (4) participants without any demographic variables (education status, marital status, household income, BMI, smoking, drinking, hypertension and diabetes), *n* = 1959. Ultimately, 16,349 participants were included (Figure 1).

### Definition of COPD

COPD diagnosis in this study relied on lung function tests, participant self-reporting confirmed by a physician, and medication information. Over three data cycles from 2007 to 2012, patients with forced expiratory volume in 1 s/ forced vital capacity (FEV1/FVC) ratios below 0.7 after inhaling bronchodilators were classified as COPD-positive (28, 29). In other cycles, the diagnosis of COPD was confirmed if the participant responded positively to questions about physician-diagnosed COPD or emphysema (“Have you ever been told that you have COPD or emphysema?”). Participants who answered “yes” were categorized as COPD-positive, while those who answered “no” were categorized as Non-COPD (30). Participants were also defined as COPD-positive when they answered positively for chronic bronchitis and also met the following criteria: history of smoking, use of specific medications such as selective phosphodiesterase 4 inhibitors, mast cell stabilizers, leukotriene modulators, and inhaled corticosteroids (31, 32).

### Definition of NHHR

Serum samples were collected from the subjects, and total cholesterol (TC) and HDL-C were measured using a series of enzymatic reactions. According to relevant studies, the NHHR is calculated as the ratio of non-HDL-C to HDL-C, where non-HDL-C is determined by subtracting HDL-C from TC and includes LDL-C and residual cholesterol. NHHR as a continuous variable was divided into tertiles for more detailed analysis. Tertile1 (lowest) for NHHR ≤ 2.21, Tertile2 (middle) for NHHR > 2.21, ≤3.37, and Tertile3 (highest) for NHHR > 3.7.

### Mortality data collection

We collected NHANES data from 2007 through 2016 and prospectively correlated them with National Death Index (NDI) mortality data.^1^ All-cause mortality refers to deaths from all causes, including death of heart, malignant neoplasms, chronic lower respiratory diseases, accidents (unintentional injuries), cerebrovascular diseases, Alzheimer’s disease, diabetes mellitus, Influenza, pneumonia, nephritis, nephrotic syndrome, nephrosis, and all other causes (residual); Respiratory mortality refers to deaths from chronic lower respiratory diseases; Cardiovascular mortality refers to deaths from heart; Tumor mortality refers to deaths from malignant neoplasms. Participants with a death status of 1 (MORTSTAT = 1) were considered to be end-point events, and the remainder were considered to be censored.

### Covariates

The covariates of the included participants, including age (≤60, >60); gender (male or female); race (Mexican American, non-Hispanic White, non-Hispanic Black, other Hispanic, or other race); socioeconomic characteristics of the participants, including education level (high school or below, some college or AA degree, college graduate or above); family income (rich, average, poor); marital status (Married/Living with partner, Widowed/divorced/separated, Never married); body mass index (BMI) (<25.0, 25.0–30.0, ≥30 kg/m^2^); lifestyle of the participants, including smoking (Yes or No); drinking alcohol (Yes or No); hypertension (Yes or No) and diabetes (Yes or No) were all analyzed to control bias. Given that 40.9% of participants lacked accessible medication information, Lipid-Lowering Medications (Yes, No or unknown) was included only as a covariate in sensitivity analyses to assess its overall impact on outcomes. The measurement procedures for these variables can be found on the CDC NHANES website at https://www.cdc.gov/nchs/nhanes. Detailed definitions of all variables can be found in the Supplementary Table 1.

### Statistical analysis

Our study utilized the weighting, stratification, and clustering methods recommended by NHANES to ensure the national representativeness of the data. The weights for this study were based on the 2-year weighting variable for laboratory examination samples (WTMEC2YR), and new weights of 1/5 × WTMEC2YR were calculated for weighted analyses (aggregated from five datasets from 2007 to 2016). Categorical variables were presented as frequencies and weighted percentages. Comparisons of categorical variables between groups were conducted using weighted chi-square tests.

Weighted multivariate logistic regression analysis was employed to investigate the association between NHHR and the prevalence of COPD during the analysis. The results were reported as odds ratio (OR) along with their corresponding 95% confidence interval (CI). Weighted Cox regression analysis was utilized to assess the correlation between NHHR use and all-cause mortality in individuals with COPD, with the results expressed as hazard ratios (HR) along with their respective 95% confidence intervals. Three models adjusting for covariates were utilized in the regression analysis for this study. Model 1 excluding covariates. Model 2 primarily corrects for demographic characteristics such as age, gender, race, educational level, marital status, and family income. Model 3 was further adjusted for all covariates, including age, gender, race, educational level, marital status, family income, BMI, smoking, drinking, hypertension, and diabetes. Kaplan-Meier curves were employed to illustrate differences in survival among the NHHR groups of COPD participants, with log-rank tests used for comparisons. A weighted Cox proportional hazard model was employed to investigate the relationship between NHHR and mortality. Subsequent subgroup analyses were conducted to explore potential interactions. RCS analyses were also carried out to assess any nonlinear effects between variables. Sensitivity analyses were performed to validate the robustness of the results.

All statistical analyses were performed using R software (version 4.4.1, R Foundation for Statistical Computing, Vienna, Austria), with a significance level set at two sides *P* < 0.05.

## Results

### Basic characteristics of participants

This study included 6,349 participants (1,271 COPD patients and 5,078 controls) with complete variables and follow-up information according to strict inclusion and exclusion criteria from 2006 to 2017. After weighted analysis, participants represented 10948908 COPD patients and 44374254 non-COPD in the entire United States population. Baseline characteristics of all participants stratified by COPD status are shown in Table 1. All data have been weighted to be representative of the entire United States population. Among all participants, the proportion of males (47.08%) was slightly smaller than that of females (52.92%), but the proportion of males with COPD (53.20%) was significantly higher than that of females (46.80%). About a quarter (25.65%) of the participants were over 60 years old, but about half (42.87%) of the patients with COPD were older than 60 years old. In addition, in the preliminary analysis, COPD is more likely to occur in the group Non-Hispanic White people, lower education level, Widowed/divorced/separated, lower family income, higher BMI, smoking, drinking habits, and comorbidities of hypertension or diabetes (*P* < 0.001). Compared with the low and medium levels of NHHR (Tertile1/2), COPD patients accounted for 33.06% in the high-level NHHR group (Tertile3), which was significantly higher than the proportion of all participants (27.33%). In addition, the proportion of all-cause deaths in COPD patients (18.38%) was significantly higher than that in all participants (6.07%).

**TABLE 1:** Weighted characteristics of the subjects in the NHANES 2007–2016 study based on the prevalence of COPD.

Characteristics, *n* (%)	Total (*n* = 6349)	Non-COPD (*n* = 5078)	COPD (*n* = 1271)	*P*
Gender				<0.001
Male	2909 (47.08)	2196 (45.58)	713 (53.20)	
Female	3440 (52.92)	2882 (54.42)	558 (46.80)	
Age				<0.001
≤60	4398 (74.35)	3791 (78.60)	607 (57.13)	
>60	1951 (25.65)	1287 (21.40)	664 (42.87)	
Race				<0.001
Mexican American	966 (8.61)	886 (10.17)	80 (2.32)	
Other Hispanic	628 (5.18)	549 (5.94)	79 (2.14)	
Non-Hispanic White	2660 (68.07)	1841 (64.25)	819 (83.53)	
Non-Hispanic Black	1206 (9.95)	986 (10.65)	220 (7.11)	
Other race	889 (8.19)	816 (9.00)	73 (4.89)	
Educational level				<0.001
High school or below	2588 (31.60)	1962 (29.35)	626 (40.72)	
Some college or AA degree	1911 (31.71)	1515 (31.55)	396 (32.35)	
College graduate or above	1850 (36.69)	1601 (39.10)	249 (26.93)	
Marital status				<0.001
Married/Living with partner	3941 (66.63)	3188 (66.76)	753 (66.08)	
Widowed/divorced/separated	1272 (16.64)	879 (14.46)	393 (25.48)	
Never married	1136 (16.73)	1011 (18.77)	125 (8.44)	
Family income				<0.001
Low	1794 (19.72)	1296 (18.54)	498 (24.50)	
Middle	2182 (32.53)	1776 (32.13)	406 (34.16)	
High	2373 (47.75)	2006 (49.33)	367 (41.33)	
BMI				0.006
≤25	1899 (30.80)	1569 (32.10)	330 (25.56)	
>25, ≤30	2062 (33.48)	1632 (32.84)	430 (36.08)	
>30	2388 (35.71)	1877 (35.06)	511 (38.36)	
Drinking				<0.001
Yes	4162 (72.46)	3130 (69.62)	1032 (84.00)	
No	2187 (27.54)	1948 (30.38)	239 (16.00)	
Smoking				<0.001
Yes	1311 (20.83)	256 (5.50)	1055 (82.98)	
No	5038 (79.17)	4822 (94.50)	216 (17.02)	
Hypertension				<0.001
Yes	1229 (15.84)	906 (14.57)	323 (20.98)	
No	5120 (84.16)	4172 (85.43)	948 (79.02)	
Diabetes				<0.001
Yes	838 (9.79)	590 (8.46)	248 (15.18)	
No	5511 (90.21)	4488 (91.54)	1023 (84.82)	
NHHR				<0.001
Tertile1	2379 (38.50)	1960 (40.00)	419 (32.46)	
Tertile2	2166 (34.17)	1726 (34.09)	440 (34.49)	
Tertile3	1804 (27.33)	1392 (25.91)	412 (33.06)	
All-cause mortality				<0.001
Yes	509 (6.07)	189 (3.03)	320 (18.38)	
No	5840 (93.93)	4889 (96.97)	951 (81.62)	

NHHR, non-high-density lipoprotein cholesterol to high-density lipoprotein cholesterol ratio; COPD, chronic obstructive pulmonary disease; BMI, body mass index.

Over an average follow-up period of 93 months, 320 participants with COPD died among 1271 COPD patients (Table 2). In primary analyses, sex, race, and BMI were not associated with mortality (*P* > 0.05). In this analysis, sociodemographic and behavioral factors associated with COPD prevalence—including Non-Hispanic White ethnicity, lower educational attainment, widowed/divorced/separated marital status, lower family income, smoking history, alcohol, and cardiometabolic comorbidities (hypertension/diabetes)—were also significantly associated with all-cause mortality in individuals with COPD. However, contrary to the proportion in all participants, the proportion of high levels of NHHR (Tertile3) in COPD deaths was significantly lower than that in all COPD patients (27.90% vs 33.06%, *P* = 0.004).

**TABLE 2:** Weighted characteristics of the subjects with COPD in the NHANES 2007–2016 study based on the all-cause mortality.

Characteristic, *n* (%)	Total (*n* = 1271)	Alive (*n* = 951)	Dead (*n* = 320)	*P*
Gender				0.224
Male	713 (53.20)	508 (52.37)	205 (56.87)	
Female	558 (46.80)	443 (47.63)	115 (43.13)	
Age				<0.001
≤60	607 (57.13)	546 (64.77)	61 (23.20)	
>60	664 (42.87)	405 (35.23)	259 (76.80)	
Race				0.293
Mexican American	80 (2.32)	71 (2.59)	9 (1.15)	
Other Hispanic	79 (2.14)	67 (2.29)	12 (1.52)	
Non-Hispanic white	819 (83.53)	594 (83.27)	225 (84.67)	
Non-Hispanic black	220 (7.11)	163 (6.80)	57 (8.49)	
Other race	73 (4.89)	56 (5.05)	17 (4.17)	
Educational level				0.002
High school or below	626 (40.72)	439 (38.52)	187 (50.49)	
Some college or AA degree	396 (32.35)	301 (31.92)	95 (34.22)	
College graduate or above	249 (26.93)	211 (29.55)	38 (15.29)	
Marital status				<0.001
Married/living with partner	753 (66.08)	591 (68.37)	162 (55.88)	
Widowed/divorced/separated	393 (25.48)	253 (22.19)	140 (40.12)	
Never married	125 (8.44)	107 (9.44)	18 (4.00)	
Family income				<0.001
Low	498 (24.50)	326 (20.18)	172 (43.72)	
Middle	406 (34.16)	307 (34.89)	99 (30.95)	
High	367 (41.33)	318 (44.94)	49 (25.33)	
BMI				0.596
≤25	330 (25.56)	234 (25.02)	96 (27.96)	
>25, ≤30	430 (36.08)	322 (36.78)	108 (32.94)	
>30	511 (38.36)	395 (38.20)	116 (39.10)	
Drinking				0.003
Yes	1032 (84.00)	788 (85.81)	244 (75.94)	
No	239 (16.00)	163 (14.19)	76 (24.06)	
Smoking				0.198
Yes	1055 (82.98)	771 (82.27)	284 (86.12)	
No	216 (17.02)	180 (17.73)	36 (13.88)	
Hypertension				0.001
Yes	323 (20.98)	219 (18.93)	104 (30.09)	
No	948 (79.02)	732 (81.07)	216 (69.91)	
Diabetes				<0.001
Yes	248 (15.18)	167 (13.00)	81 (24.87)	
No	1023 (84.82)	784 (87.00)	239 (75.13)	
NHHR				0.051
1	676 (51.47)	489 (49.87)	187 (58.59)	
2	595 (48.53)	462 (50.13)	133 (41.41)	
NHHR				0.004
Tertile1	419 (32.46)	286 (30.20)	133 (42.46)	
Tertile2	440 (34.49)	347 (35.58)	93 (29.65)	
Tertile3	412 (33.06)	318 (34.22)	94 (27.90)	

NHHR, non-high-density lipoprotein cholesterol to high-density lipoprotein cholesterol ratio; COPD, chronic obstructive pulmonary disease; BMI, body mass index.

### Associations between NHHR and COPD prevalence

**FIGURE 2:**
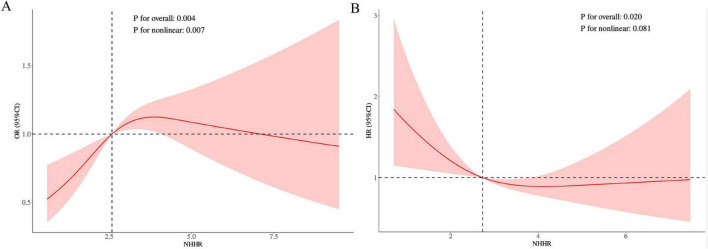
RCS analysis of NHHR for COPD prevalence **(A)** and COPD all-cause mortality **(B)**.

This study initially analyzed the impact of NHHR levels on the prevalence of COPD in all participants (Table 3). In a fully adjusted weighted logistic regression model, the highest NHHR level significantly increased the risk of COPD prevalence by 62% compared to the lowest NHHR level (Model 3: OR = 1.62, 95% CI: 1.11–2.39, *P* = 0.017). Specifically, in both weighted logistic regression models with no covariate adjustment and partial covariate adjustment, the highest NHHR level significantly increased the risk of COPD development compared to the lowest NHHR level (Model 1: OR = 1.57, 95% CI: 1.23–2.01, *P* < 0.001; Model 2: OR = 1.54, 95% CI: 1.19–2.01, *P* < 0.001). Moreover, when NHHR was included as a continuous variable in a weighted regression model, it remained a risk factor for COPD development in regression models without covariate adjustment and those adjusted for some covariates (Model 1: OR = 1.13, 95% CI: 1.07–1.19, *P* < 0.001; Model 2: OR = 1.13, 95% CI: 1.06–1.20, *P* = 0.002). To better understand the relationship between NHHR and COPD, RCS was utilized to explore the nonlinear relationship. RCS results, after adjusting for all covariates, indicated a nonlinear relationship between NHHR and COPD (*P* for nonlinear = 0.007). The curve exhibited an inverted L shape (Figure 2A), suggesting that within a certain range, an increase in NHHR led to a gradual rise in the risk of COPD until reaching a point where no statistical difference was observed. This finding was also supported by the regression model.

**TABLE 3:** Association between NHHR and COPD prevalence or mortality.

Variables	Model 1		Model 2		Model 3	
	**OR/HR (95%CI)**	***P***	**OR/HR (95%CI)**	***P***	**OR/HR (95%CI)**	***P***
**COPD prevalence**						
NHHR (continuous)	1.13 (1.07 ∼ 1.19)	<0.001	1.13 (1.06 ∼ 1.20)	0.002	1.08 (0.97 ∼ 1.21)	0.173
**NHHR**						
Tertile1	1.00 (Reference)		1.00 (Reference)		1.00 (Reference)	
Tertile2	1.25 (1.00 ∼ 1.55)	0.051	1.25 (1.00 ∼ 1.57)	0.051	1.43 (1.07 ∼ 1.82)	0.014
Tertile3	1.57 (1.23 ∼ 2.01)	<0.001	1.54 (1.19 ∼ 2.01)	0.002	1.62 (1.11 ∼ 2.39)	0.017
*P* for trend		<0.001		0.002		0.023
**COPD mortality**						
NHHR (continuous)	0.82 (0.72–0.93)	0.002	0.89 (0.79–0.99)	0.037	0.89 (0.80–0.99)	0.027
**NHHR**						
Tertile1	1.00 (Reference)		1.00 (Reference)		1.00 (Reference)	
Tertile2	0.59 (0.41–0.85)	0.004	0.78 (0.55–1.09)	0.149	0.77 (0.55–1.08)	0.130
Tertile3	0.55 (0.41–0.75)	<0.001	0.75 (0.56–1.01)	0.055	0.74 (0.56–0.98)	0.037
*P* for trend		<0.001		0.039		0.031

Model 1: no covariates were adjusted. Model 2: gender, age, race, educational level, marital status and family income were adjusted. Model 3: gender, age, race, educational level, marital status, family income, BMI, smoking, drinking, hypertension and diabetes were adjusted. NHHR, non-high-density lipoprotein cholesterol to high-density lipoprotein cholesterol ratio; COPD, chronic obstructive pulmonary disease; OR, odds ratio; HR, hazard ratio; CI, confidence interval.

### Associations between NHHR and COPD all-cause mortality

**FIGURE 3:**
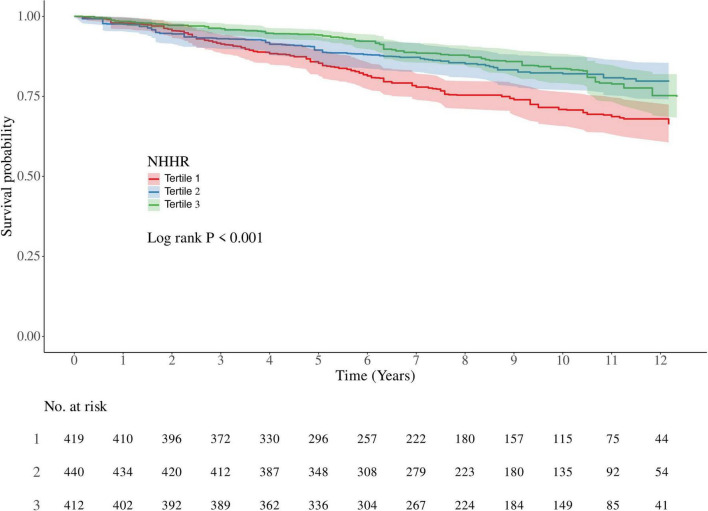
Kaplan–Meier analysis of all-cause mortality in participants with COPD.

We then included only participants defined as having COPD and analyzed NHHR levels in relation to all-cause mortality in participants with COPD (Table 3). Unexpectedly, NHHR levels showed a significant negative association with all-cause mortality in COPD patients in all models. In a weighted Cox regression model fully adjusted for covariates, the highest level of NHHR significantly reduced the risk of all-cause mortality by 26% compared to the lowest level of NHHR (Model 3: HR = 0.74, 95% CI: 0.56–0.98, *P* = 0.037). Similarly, NHHR served as a protective factor for all-cause mortality in a fully adjusted covariate-weighted Cox regression model with NHHR as a continuous variable (Model 3: HR = 0.89, 95% CI: 0.880–0.99, *P* = 0.027). In weighted Kaplan-Meier survival analyses, adjusting for all covariates, the high-level NHHR group had the highest survival rate at the same time point, the medium-level NHHR group had a survival rate in the middle of the three, and the low-level NHHR group (Tertile2) had the lowest survival rate (Figure 3). The RCS model adjusting for all covariates was similarly used to assess the association of NHHR with COPD all-cause mortality. RCS results showed a positive linear relationship (*P* for nonlinear = 0.081) (Figure 2B). The curve was positively L-shaped, indicating that increased NHHR was associated with reduced mortality to a certain extent.

### Subgroup analyses

**FIGURE 4:**
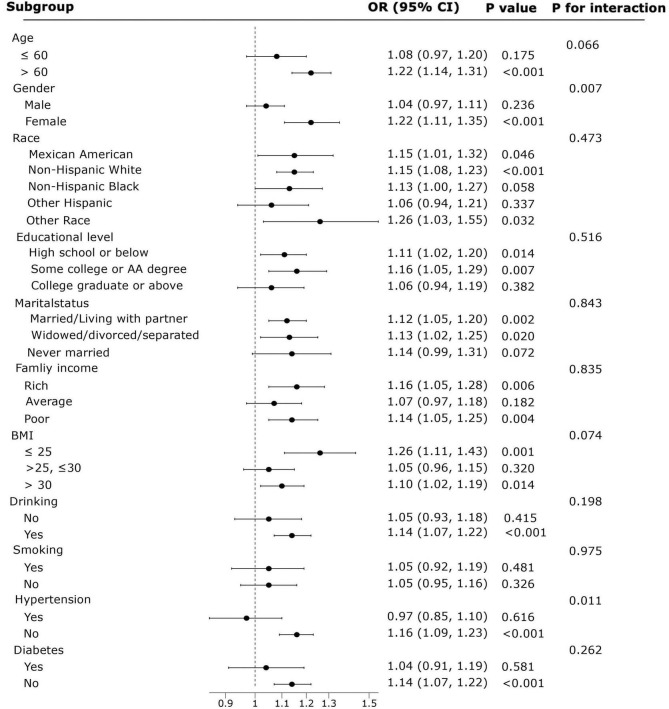
Forest plot showing the results of subgroup analysis of the associations between NHHR and COPD prevalence.

**FIGURE 5:**
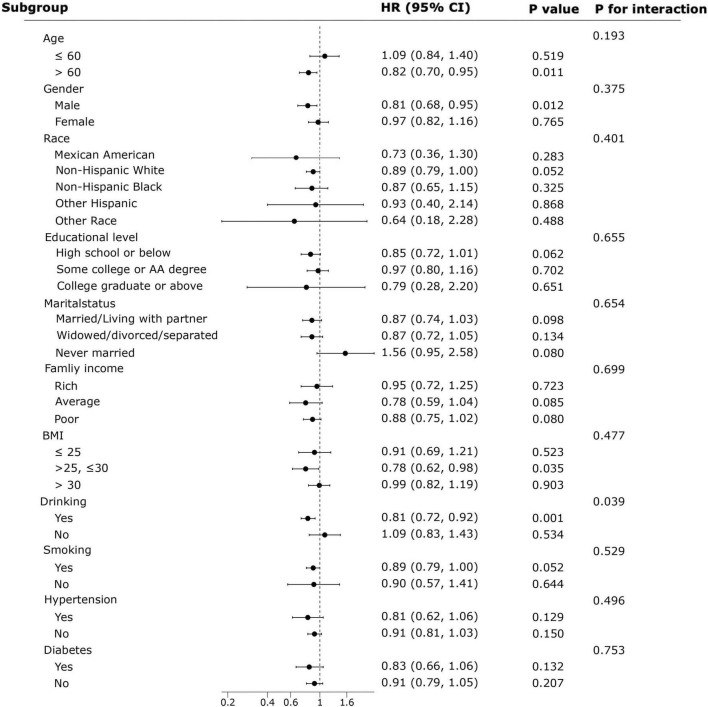
Forest plot showing the results of subgroup analysis of the associations between NHHR and COPD all-cause mortality.

This study examined the associations between NHHR and the prevalence of COPD (Figure 4) and all-cause mortality (Figure 5) in various subgroups. Age, gender, race, educational level, marital status, family income, BMI, smoking, drinking, hypertension, and diabetes were included as stratification factors in the subgroup analyses.

In the subgroup analyses of NHHR and COPD prevalence, factors other than gender and hypertension status did not exhibit significant interactions with NHHR and COPD prevalence (*P* > 0.05 for interaction), which supports the reliability of our results. High NHHR levels were more closely linked to COPD prevalence in women compared to men (OR = 1.22, 95% CI: 1.11–1.35, *P* < 0.001, *P* for interaction = 0.007). Additionally, subgroups such as those over age 60, non-Hispanic white people, individuals with less than a college degree, unmarried individuals, those with non-moderate income, individuals with high or low BMI, and alcohol consumers were more susceptible to the effects of the NHHR.

In the subgroup analyses of NHHR and COPD all-cause mortality, factors other than alcohol drinking habit did not display significant interactions with NHHR and COPD prevalence (*P* for interactions >0.05), supporting the robustness of our mortality analyses.

### Sensitivity analyses

When the patients were divided into high and low groups according to the median NHHR, the trend of the effect of NHHR on the prevalence and mortality of COPD remained consistent (Supplementary Table 2). In the weighted logistic regression model fully adjusted for all covariates, the high NHHR level group showed a 46% increased risk of COPD compared to the low NHHR level group (Model 3: OR = 1.46, 95%CI = 1.10–1.94, *P* = 0.010). In the mortality analysis, a high level of NHHR was associated with a decreased risk of all-cause mortality in COPD patients (Model 1: OR = 0.70, 95%CI = 0.51–0.96, *P* = 0.027). Weighted Kaplan-Meier analysis also indicated that the high NHHR group had a higher survival rate than the low NHHR group (log-rank *P* = 0.020) (Supplementary Figure 1A). The results of the weighted Kaplan-Meier analyses remained consistent with the previous analyses when participants who had been followed for less than 3 years were excluded (Supplementary Figure 1B).

Furthermore, a separate analysis of participants from 2007 to 2012, based on lung function criteria, yielded results consistent with the overall 10-year study results (Supplementary Table 3). COPD status, defined based on self-reported questionnaire data, medication use and smoking history from 2013 to 2016, also demonstrated consistent associations with the outcomes, aligning with the primary findings of the overall analysis (Supplementary Table 4).

Notably, lipid-lowering medications may confound the association between NHHR and COPD-related endpoints. However, sensitivity analyses adjusting for these medications yielded results concordant with those in the total population, suggesting minimal impact of lipid-lowering drug use on the observed relationships (Supplementary Table 5).

To assess the robustness of the association between NHHR and all-cause mortality, we conducted sensitivity analyses examining cause-specific mortality endpoints in COPD patients. We evaluated NHHR’s associations with respiratory mortality, cardiovascular mortality, and tumor mortality (Supplementary Table 6). Notably, NHHR demonstrated a statistically significant association with respiratory mortality (Model 3: HR = 0.77, 95% CI: 0.63–0.94, *P* = 0.009), whereas no significant associations were observed with cardiovascular mortality (Model 3: HR = 0.91, 95% CI: 0.68–1.22, *P* = 0.525) or tumor mortality (Model 3: HR = 0.96, 95% CI: 0.80–1.16, *P* = 0.696).

## Discussion

In this NHANES cross-sectional analysis, higher NHHR levels showed a positive association with COPD prevalence in the general population. Stratified analyses revealed a contrasting pattern in COPD mortality outcomes: lower NHHR levels were correlated with elevated all-cause mortality risk among COPD patients, while higher levels appeared inversely related to mortality. These differential associations suggest that NHHR’s role may vary across COPD stages—potentially reflecting distinct biological pathways during disease initiation versus progression. Given the observational design, future studies will use a multi-dimensional staging system (integrating GOLD grading, imaging emphysema score, and plasma inflammatory markers) to accurately analyze the prognostic heterogeneity of NHHR.

LDL is the primary component of non-HDL-C, and an increase in NHHR indicates a higher proportion of LDL. This study suggests that elevated NHHR levels are associated with higher COPD prevalence. This association persisted when we adjusted for the use of lipid-lowering drugs as a covariate, indicating that the association was not affected by lipid-lowering drugs. Some studies have indicated that the mean serum LDL level in COPD patients was significantly higher compared to the control group (18.62 ±7.56 vs 12.57 ± 5.90 mU/L, *P* < 0.05); the serum LDL level was negatively correlated with predicted FEV1% (*r* = −0.347, *P* = 0.016) but positively correlated with C-reactive protein (CRP) and reactive oxygen species (ROS) levels (33). Oxidized LDL (ox-LDL) serves as a marker of oxidative stress. Elevated levels of oxidized LDL contribute to systemic inflammation, with an imbalance between oxidation and antioxidant levels being a contributing factor to COPD (34, 35). Furthermore, elevated blood lipid levels can increase the risk of cardiovascular and cerebrovascular diseases, as well as diabetes, all of which are independent risk factors for COPD (36). These findings align with our research on NHHR and the prevalence of COPD.

In the subgroup analysis, a significant positive association was observed between LDL-C levels and COPD in female patients, while no such association was detected in males. This gender disparity aligns with epidemiological evidence indicating that female COPD patients generally experience more severe clinical manifestations across all age groups (37). The observed difference may be mechanistically linked to estrogen’s dual regulatory roles in lipid metabolism and pulmonary pathophysiology (38). Notably, estrogens undergo metabolic conversion to catechol estrogens via hydroxylation. These metabolites subsequently participate in cytochrome P450-mediated redox cycling reactions (39, 40). This biochemical pathway may exacerbate oxidative stress burden in female smokers, potentially explaining their heightened susceptibility to COPD development compared to male counterparts. The pro-oxidant effects of estrogen derivatives could synergize with LDL-C-induced reactive oxygen species generation, creating a permissive microenvironment for lung parenchymal damage and accelerated disease progression.

In terms of the potential pathogenic mechanism, ox-LDL activates a variety of transcription factors involved in the pathogenesis of COPD, including activator protein 1, NF-κB, signal transducer and activator of transcription (STAT), and hypoxia-inducible factor 1(HIF-1) (34). Additionally, ox-LDL has been shown to increase the expression or secretion of chemokines, such as monocyte chemoattractant protein-1, macrophage inflammatory protein (MIF)-1α, and MIF-2, as well as proinflammatory cytokines, such as interleukin (IL) -1β, IL-12, and tumor necrosis factor-α (TNF-α) (35, 41). These inflammatory mediators are known to contribute to the pathogenesis of COPD. Furthermore, ox-LDL increases intracellular ROS production (42), leading to elevated oxidative stress. This may activate stress kinases and redox-sensitive transcription factors, thereby enhancing inflammation. Consequently, this signaling pathway increases the expression of a unique set of proinflammatory mediators that contribute to COPD.

Contrasting with its positive association with COPD prevalence, this cross-sectional analysis revealed an inverse association between NHHR levels and all-cause mortality among COPD patients. This pattern aligns with observations that elevated LDL-C levels – a component of NHHR – correlate with higher BMI and improved nutritional indices in chronic respiratory diseases (43, 44). Notably, BMI constitutes a key element of the BODE index, a validated prognostic tool for COPD (45). Our findings resonate with prior reports describing the “obesity paradox” in COPD, where higher BMI independently predicts better survival (45–48). The mechanisms underlying these associations may involve multiple interrelated factors. Adipose tissue preservation has been hypothesized to mitigate lung function decline through anti-inflammatory effects and energy reserve maintenance (49). Furthermore, lipolysis-induced adipose tissue atrophy precedes muscle loss (50), and inhibiting lipolysis can counteract both adipose tissue and muscle atrophy, emphasizing the importance of maintaining lipid homeostasis (51). Additionally, dyslipidemia is associated with sarcopenia (52), a condition strongly associated with COPD mortality (53). In our cohort, the inverse relationship between NHHR and mortality might reflect shared pathways involving nutritional status preservation and reduced sarcopenia risk (54).

Dietary patterns may exert dual influences on this association: sustained appetite maintenance potentially increases lipid intake (thereby elevating LDL-C) while adequate protein and caloric consumption improves nutritional status, indirectly supporting muscle mass preservation (54, 55). Emerging evidence indicates that various nutrients with anti-inflammatory properties, particularly those abundant in plant-based diets rich in vegetables, fruits, and specific vitamins, may mitigate COPD mortality risk (56). This aligns with findings from Lieke et al.’s systematic review demonstrating that anti-inflammatory dietary patterns significantly reduce circulating inflammatory markers such as CRP and IL-6 (57), potentially creating a favorable microenvironment for COPD management.

The advantages and disadvantages of this study are as follows: Firstly, it utilizes real clinical data from a nationally representative sample of the United States population, with a large sample size. Through complex sampling, weighting, and adjustment for confounding factors in the analysis, the study accurately reveals the associations of NHHR on morbidity and mortality of COPD. Furthermore, the long-term follow-up tracking of the number of deaths provides a strong foundation for the study analysis. Secondly, as a common clinical examination index, NHHR allows for cost-effective COPD prevention and disease progression monitoring, with significant potential for broad application and promotion. However, there are some shortcomings to acknowledge. Firstly, the metabolic parameters captured in this study reflect single-timepoint measurements during participant enrollment, which may not fully encapsulate the dynamic nature of lipid homeostasis or account for temporal fluctuations in biological markers. While our models adjusted for key demographic and lifestyle confounders (age, smoking status, BMI), the cross-sectional design inherently limits our ability to address chronic environmental exposures (e.g., PM2.5 accumulation over decades, intermittent occupational hazards like silica dust) that require longitudinal assessment. Particularly, multidimensional socioeconomic influences – manifesting through geographically patterned dietary habits, differential healthcare-seeking behaviors, and prescription medication adherence – may introduce residual confounding that standard statistical adjustments cannot sufficiently disentangle. This measurement temporal limitation underscores the necessity for future prospective studies incorporating repeated biomarker assessments and geospatial exposure mapping to better characterize lifetime metabolic trajectories. Secondly, the cross-sectional studies cannot make causal judgments, and we will continue to explore them in prospective studies in the future. And the clinical threshold of NHHR will be explored in future prospective cohort studies. Additionally, mendelian randomization (MR) offers a good tool for investigating causal relationships. While NHHR, as a composite biomarker, cannot be directly utilized in MR analyses, future research could leverage its individual components (e.g., HDL cholesterol) as proxies for causal inference studies in this field. Thirdly, the study cohort primarily comprised non-Hispanic White Americans, potentially limiting generalizability to other ethnic or global populations, our subgroup analyses revealed consistent associations between NHHR and COPD across racial/ethnic groups, suggesting minimal heterogeneity in these relationships. However, socioeconomic and healthcare system disparities in low- and middle-income countries may introduce context-specific modifiers not captured in this analysis. Future investigations will prioritize validation of these findings in diverse settings, including planned studies in China, to assess the framework’s applicability across resource-varying environments. Lastly, changes in diagnostic criteria, spirometry-defined (2007–2012) and questionnaire/medication-based (2013–2016) COPD diagnoses, may have led to misclassification of disease, but stratified analyses showed cross-method consistency of results, suggesting that potential bias had a small impact on the main conclusions.

## Conclusion

This cross-sectional study revealed a paradoxical association between elevated NHHR levels and COPD outcomes. While higher NHHR levels were positively associated with COPD prevalence in the general population, they were inversely correlated with all-cause mortality among COPD patients. Further longitudinal studies are warranted to explore whether lipid profile modulation could simultaneously mitigate COPD prevalence and improve post-diagnosis survival.

## Data Availability

Publicly available datasets were analyzed in this study. This data can be found here: https://www.cdc.gov/nchs/nhanes/.

## Footnotes

1
https://www.cdc.gov/nchs/data-linkage/mortality

## Supplementary material

Supplementary Figure 1 Kaplan-Meier analysis of all-cause mortality among COPD participants based on NHHR dichotomization **(A)** and excluding participants with less than 3 years of follow-up **(B)**.
