# Supplementary material for: Association of the non-high-density lipoprotein cholesterol to high-density lipoprotein cholesterol ratio (NHHR) with COPD prevalence and all-cause mortality: a population-based study based on NHANES 2007–2016
Source: Front Med (Lausanne). 2025 Apr 3;12:1533744. doi: 10.3389/fmed.2025.1533744 (PMC12003284; doi:10.3389/fmed.2025.1533744)
Supplement: Supplementary file 1 [file Data_Sheet_1.docx]

**Supplementary table**

**Table S1.** Definition of variables involved in this study.

| Variables | Description in NHANES |
| --- | --- |
| Age | Divided into two groups:  ≤ 60 years old; >60 years old |
| Gender | Male and Female |
| Race | Mexican American, Other Hispanic, Non-Hispanic Black, Non-Hispanic White, Other Race |
| Educational level | Divided into three groups: High School or below, Some college or AA, College or above. "Some college or AA" refers to the proportion of people aged 25 and older who have either taken some college courses but not earned a degree, or have an associate degree. |
| Marital status | Divided into three groups: Married/Living with partner; Widowed/divorced/separated; Never married. |
| Family income | Divided into three groups:  Rich: Annual family income greater than $65,000;  Average: Annual family income between $25,000 and $65,000;  Poor: Annual family income lower than $25,000. |
| BMI | Body Mass Index divided into three groups: ≤ 25; >25, ≤30; >30. |
| Smoking | Have you smoked 100+ cigarettes in life? Yes/No |
| Drinking | Had at least 12 alcohol drinks/1 year? Yes/No |
| Hypertension | Systolic blood pressure greater than 140 mmHg or diastolic blood pressure greater than 100 mmHg. |
| Diabetes | Doctor told you have diabetes? Yes/No |
| Lipid-Lowering Medications | Based on medication data from the NHANES database, participants with a history of using the following lipid-lowering agents were defined as lipid-lowering medication users:  1.Statins: atorvastatin, simvastatin, fluvastatin, lovastatin, pravastatin, rosuvastatin, pitavastatin  2.Cholesterol Absorption Inhibitors: ezetimibe  3.Fibrates: fenofibrate, gemfibrozil  4.Niacin: Derivatives niacin  Participants with available medication data but no use of lipid-lowering agents were defined as non-users. Additionally, those without medication records were categorized into a separate group. Given that 40.9% of participants lacked accessible medication information, lipid-lowering drug status was included only as a covariate in sensitivity analyses to assess its overall impact on outcomes. |
| NHHR | NHHR is calculated as total cholesterol (mg/dL) minus HDL cholesterol (mg/dL), then divided by HDL cholesterol (mg/dL).  Tertile1 ≤ 2.21;  Tertile2 > 2.21, ≤ 3.37;  Tertile3 >3.37. |
| COPD | In the cycle 2007-2012, COPD was defined as a ratio of forced expiratory volume in 1 second (FEV1) to forced vital capacity (FVC) of less than 0.70, as assessed by spirometry after bronchodilator use.  In the cycle 2013-2016, due to the lack of lung function data, we used the following methods to determine COPD: Participants who responded affirmatively to the query, “Have you ever been told that you have emphysema?” were classified as having COPD. Patients with COPD were also defined as participants who had a tobacco use history or chronic bronchitis, and were currently using one of the following COPD prescription medications: selective phosphodiesterase-4 inhibitors, somatic cell stabilizers, leukotriene modulators, or inhaled corticosteroids. |
| Mortality | Mortality data was determined by linking study data to the National Mortality Index through December 2019 (https://www.cdc.gov/nchs/data-linkage/mortality.htm).  All-cause mortality refers to deaths from all causes, including death of heart, malignant neoplasms, chronic lower respiratory diseases, accidents (unintentional injuries), cerebrovascular diseases, Alzheimer’s disease, diabetes mellitus, Influenza, pneumonia, nephritis, nephrotic syndrome, nephrosis, and all other causes (residual);  Respiratory mortality refers to deaths from chronic lower respiratory diseases;  Cardiovascular mortality refers to deaths from heart;  Tumor mortality refers to deaths from malignant neoplasms. |

Table S2. Association between the dichotomy of NHHR and COPD prevalence or mortality.

| Variables | Model 1 | | Model 2 | | Model 3 | |
| --- | --- | --- | --- | --- | --- | --- |
|  | OR/HR (95%CI) | P | OR/HR (95%CI) | P | OR/HR (95%CI) | P |
| COPD prevalence |  |  |  |  |  |  |
| NHHR |  |  |  |  |  |  |
| Low | 1.00 (Reference) |  | 1.00 (Reference) |  | 1.00 (Reference) |  |
| High | 1.45 (1.19 ~ 1.75) | <0.001 | 1.38 (1.13 ~ 1.68) | 0.002 | 1.46 (1.10 ~ 1.94) | 0.010 |
| COPD mortality |  |  |  |  |  |  |
| NHHR |  |  |  |  |  |  |
| Low | 1.00 (Reference) |  | 1.00 (Reference) |  | 1.00 (Reference) |  |
| High | 0.70 (0.51 - 0.96) | 0.027 | 0.86 (0.63 - 1.16) | 0.323 | 0.86 (0.65 - 1.13) | 0.280 |

Model 1: no covariates were adjusted.

Model 2: gender, age, race, educational level, marital status, and family income were adjusted.

Model 3: gender, age, race, educational level, marital status, family income, BMI, smoking, drinking, hypertension and diabetes were adjusted.

Abbreviation: NHHR, non-high-density lipoprotein cholesterol to high-density lipoprotein cholesterol ratio; COPD, chronic obstructive pulmonary disease; OR, odds ratio; HR, hazard ratio; CI, confidence interval.

Table S3. Association between NHHR and COPD prevalence or COPD all-cause mortality in 2007-2012.

| Variables | Model 1 | | Model 2 | | Model 3 | |
| --- | --- | --- | --- | --- | --- | --- |
|  | OR/HR (95%CI) | *P* | OR/HR (95%CI) | *P* | OR/HR (95%CI) | *P* |
| **COPD prevalence** |  |  |  |  |  |  |
| NHHR (continuous) | 1.08 (0.96 ~ 1.22) | 0.190 | 1.14 (0.99 ~ 1.33) | 0.076 | 1.07 (0.92 ~ 1.25) | 0.373 |
| NHHR |  |  |  |  |  |  |
| Tertile1 | 1.00 (Reference) |  | 1.00 (Reference) |  | 1.00 (Reference) |  |
| Tertile2 | 1.17 (0.91 ~ 1.51) | 0.215 | 1.37 (1.04 ~ 1.82) | 0.025 | 1.32 (0.99 ~ 1.75) | 0.056 |
| Tertile3 | 1.49 (0.99 ~ 2.25) | 0.059 | 1.89 (1.20 ~ 2.99) | 0.008 | 1.69 (1.03 ~ 2.75) | 0.037 |
| *P* for trend |  | 0.065 |  | 0.012 |  | 0.005 |
| **All-cause mortality of COPD** |  |  |  |  |  |  |
| NHHR (continuous) | 0.88 (0.77 ~ 0.99) | 0.040 | 0.94 (0.84 ~ 1.06) | 0.334 | 0.88 (0.79 ~ 0.98) | 0.045 |
| NHHR |  |  |  |  |  |  |
| Tertile1 | 1.00 (Reference) |  | 1.00 (Reference) |  | 1.00 (Reference) |  |
| Tertile2 | 0.64 (0.46 ~ 0.89) | 0.007 | 0.84 (0.62 ~ 1.15) | 0.278 | 0.79 (0.58 ~ 1.07) | 0.125 |
| Tertile3 | 0.52 (0.34 ~ 0.79) | 0.002 | 0.69 (0.45 ~ 1.04) | 0.076 | 0.65 (0.43 ~ 0.98) | 0.040 |
| *P* for trend |  | 0.008 |  | 0.221 |  | 0.016 |

Model 1: no covariates were adjusted.

Model 2: gender, age, race, educational level, marital status, and family income were adjusted.

Model 3: gender, age, race, educational level, marital status, family income, BMI, smoking, drinking, hypertension and diabetes were adjusted.

Abbreviation: NHHR, non-high-density lipoprotein cholesterol to high-density lipoprotein cholesterol ratio; COPD, chronic obstructive pulmonary disease; OR, odds ratio; HR, hazard ratio; CI, confidence interval.

Table S4. Association between NHHR and COPD prevalence or COPD all-cause mortality in 2013-2016.

| Variables | Model 1 | | Model 2 | | Model 3 | |
| --- | --- | --- | --- | --- | --- | --- |
|  | OR/HR (95%CI) | *P* | OR/HR (95%CI) | *P* | OR/HR (95%CI) | *P* |
| **COPD prevalence** |  |  |  |  |  |  |
| NHHR (continuous) | 1.07 (0.98 ~ 1.16) | 0.136 | 1.10 (1.00 ~ 1.22) | 0.059 | 1.25 (1.01 ~ 1.55) | 0.038 |
| NHHR |  |  |  |  |  |  |
| Tertile1 | 1.00 (Reference) |  | 1.00 (Reference) |  | 1.00 (Reference) |  |
| Tertile2 | 1.08 (0.70 ~ 1.65) | 0.722 | 1.08 (0.70 ~ 1.68) | 0.698 | 2.85 (1.17 ~ 6.95) | 0.025 |
| Tertile3 | 1.13 (0.77 ~ 1.65) | 0.521 | 1.18 (0.78 ~ 1.80) | 0.406 | 3.04 (1.26 ~ 7.31) | 0.018 |
| *P* for trend |  | 0.703 |  | 0.563 |  | 0.044 |
| **All-cause mortality of COPD** |  |  |  |  |  |  |
| NHHR (continuous) | 0.72 (0.52 ~ 0.99) | 0.042 | 0.73 (0.55 ~ 0.96) | 0.025 | 0.73 (0.58 ~ 0.93) | 0.011 |
| NHHR |  |  |  |  |  |  |
| Tertile1 | 1.00 (Reference) |  | 1.00 (Reference) |  | 1.00 (Reference) |  |
| Tertile2 | 0.87 (0.42 ~ 1.80) | 0.722 | 0.82 (0.45 ~ 1.50) | 0.523 | 0.98 (0.55 ~ 1.73) | 0.937 |
| Tertile3 | 0.35 (0.16 ~ 0.75) | 0.007 | 0.44 (0.20 ~ 0.93) | 0.031 | 0.43 (0.20 ~ 0.91) | 0.027 |
| *P* for trend |  | 0.019 |  | 0.042 |  | 0.046 |

Model 1: no covariates were adjusted.

Model 2: gender, age, race, educational level, marital status, and family income were adjusted.

Model 3: gender, age, race, educational level, marital status, family income, BMI, smoking, drinking, hypertension and diabetes were adjusted.

Abbreviation: NHHR, non-high-density lipoprotein cholesterol to high-density lipoprotein cholesterol ratio; COPD, chronic obstructive pulmonary disease; OR, odds ratio; HR, hazard ratio; CI, confidence interval.

Table S5. Association between NHHR and COPD prevalence or COPD all-cause mortality adjusting for lipid-lowering medications in 2007-2016.

| Variables | **COPD prevalence**  (Model 3) | | **All-cause mortality of COPD** (Model 3) | |
| --- | --- | --- | --- | --- |
|  | OR (95%CI) | *P* | HR (95%CI) | *P* |
|  |  |  |  |  |
| NHHR (continuous) | 1.15 (1.03 ~ 1.28) | 0.014 | 0.90 (0.83 ~ 0.97) | 0.006 |
| NHHR |  |  |  |  |
| Tertile1 | 1.00 (Reference) |  | 1.00 (Reference) |  |
| Tertile2 | 1.52 (1.15 ~ 2.02) | 0.004 | 0.77 (0.60 ~ 0.99) | 0.044 |
| Tertile3 | 2.08 (1.40 ~ 3.07) | <0.001 | 0.75 (0.58 ~ 0.96) | 0.024 |
| *P* for trend |  | <0.001 |  | 0.024 |

Model 3: Gender, age, race, educational level, marital status, family income, BMI, smoking, drinking, hypertension, diabetes and lipid-lowering medications were adjusted.

Abbreviation: NHHR, non-high-density lipoprotein cholesterol to high-density lipoprotein cholesterol ratio; COPD, chronic obstructive pulmonary disease; OR, odds ratio; HR, hazard ratio; CI, confidence interval.

Table S6. Association between NHHR and respiratory mortality, cardiovascular mortality and tumor mortality in COPD.

| Variables | Model 1 | | Model 2 | | Model 3 | |
| --- | --- | --- | --- | --- | --- | --- |
|  | HR (95%CI) | *P* | HR (95%CI) | *P* | HR (95%CI) | *P* |
| **Respiratory mortality** |  |  |  |  |  |  |
| NHHR (continuous) | 0.67 (0.51 ~ 0.88) | 0.005 | 0.75 (0.60 ~ 0.95) | 0.018 | 0.77 (0.63 ~ 0.94) | 0.009 |
| NHHR |  |  |  |  |  |  |
| Tertile1 | 1.00 (Reference) |  | 1.00 (Reference) |  | 1.00 (Reference) |  |
| Tertile2 | 0.55 (0.26 ~ 1.17) | 0.119 | 0.83 (0.40 ~ 1.71) | 0.606 | 0.87 (0.42 ~ 1.80) | 0.708 |
| Tertile3 | 0.44 (0.16~ 1.22) | 0.114 | 0.70 (0.27 ~ 1.80) | 0.463 | 0.76 (0.35 ~ 1.63) | 0.479 |
| *P* for trend |  | 0.534 |  | 0.128 |  | 0.512 |
| **Cardiovascular** **mortality** |  |  |  |  |  |  |
| NHHR (continuous) | 0.91 (0.67~ 1.25) | 0.563 | 0.91 (0.67~ 1.25) | 0.565 | 0.91 (0.68 ~ 1.22) | 0.525 |
| NHHR |  |  |  |  |  |  |
| Tertile1 | 1.00 (Reference) |  | 1.00 (Reference) |  | 1.00 (Reference) |  |
| Tertile2 | 0.66 (0.30 ~ 1.45) | 0.298 | 0.75 (0.33 ~ 1.70) | 0.497 | 0.72 (0.34 ~ 1.52) | 0.395 |
| Tertile3 | 0.75 (0.35~ 1.58) | 0.449 | 0.78 (0.36 ~ 1.69) | 0.526 | 0.74 (0.36 ~ 1.52) | 0.441 |
| *P* for trend |  | 0.421 |  | 0.449 |  | 0.442 |
| **Tumor mortality** |  |  |  |  |  |  |
| NHHR (continuous) | 0.08 (0.74 ~ 1.06) | 0.184 | 0.96 (0.81 ~ 1.14) | 0.635 | 0.96 (0.80 ~ 1.16) | 0.696 |
| NHHR |  |  |  |  |  |  |
| Tertile1 | 1.00 (Reference) |  | 1.00 (Reference) |  | 1.00 (Reference) |  |
| Tertile2 | 0.49 (0.26 ~ 0.94) | 0.032 | 0.60 (0.32 ~ 1.12) | 0.108 | 0.58 (0.30 ~ 1.12) | 0.102 |
| Tertile3 | 0.62 (0.36 ~ 1.07) | 0.087 | 0.81 (0.47 ~ 1.41) | 0.458 | 0.78 (0.41 ~ 1.49) | 0.457 |
| *P* for trend |  | 0.088 |  | 0.551 |  | 0.412 |

Model 1: no covariates were adjusted.

Model 2: gender, age, race, educational level, marital status, and family income were adjusted.

Model 3: gender, age, race, educational level, marital status, family income, BMI, smoking, drinking, hypertension and diabetes were adjusted.

Abbreviation: NHHR, non-high-density lipoprotein cholesterol to high-density lipoprotein cholesterol ratio; COPD, chronic obstructive pulmonary disease; HR, hazard ratio; CI, confidence interval.
