# Supplementary figures and images for: Association of the non-high-density lipoprotein cholesterol to high-density lipoprotein cholesterol ratio (NHHR) with COPD prevalence and all-cause mortality: a population-based study based on NHANES 2007–2016
Source: Front Med (Lausanne). 2025 Apr 3;12:1533744. doi: 10.3389/fmed.2025.1533744 (PMC12003284; doi:10.3389/fmed.2025.1533744)

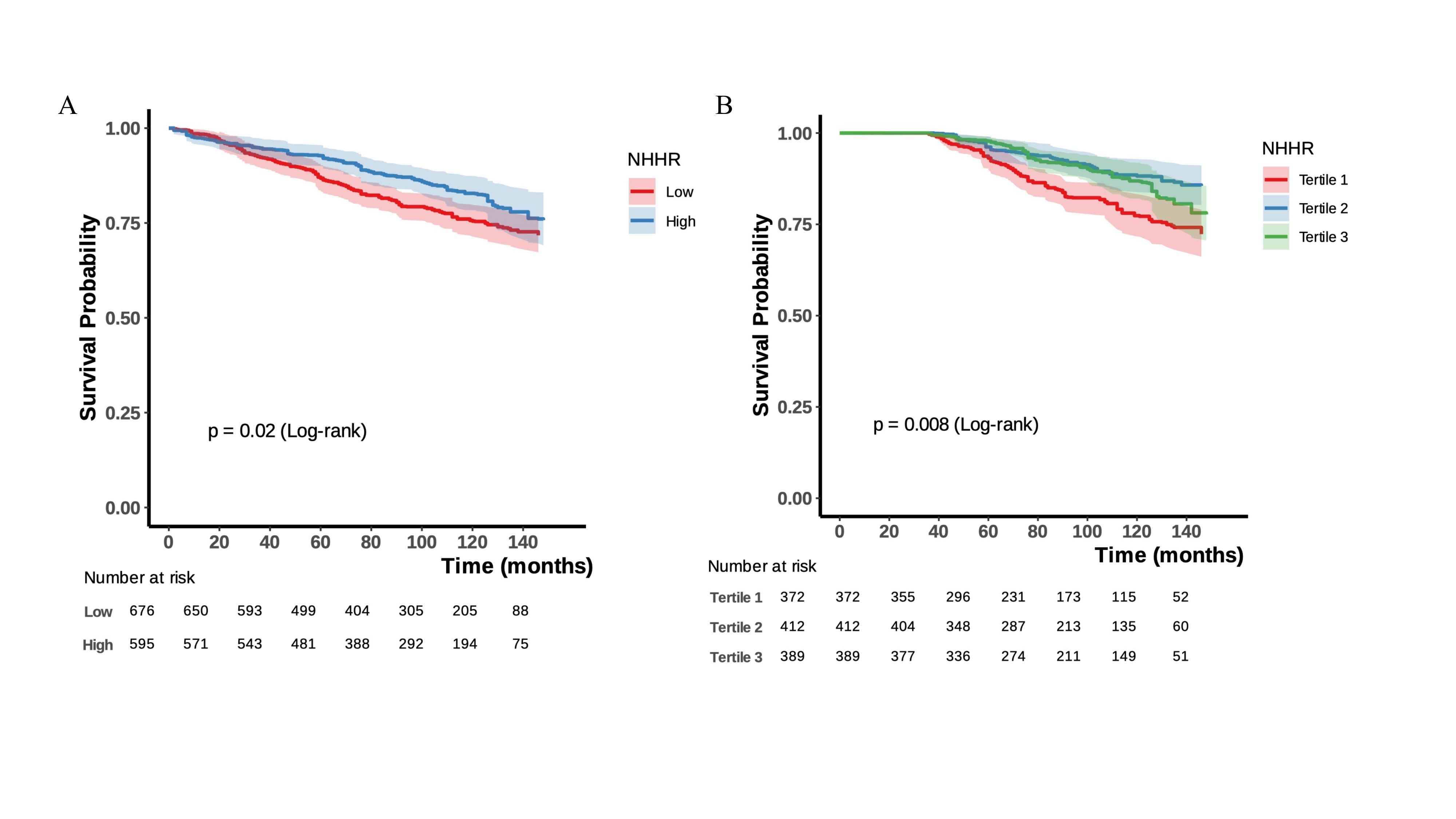

Supplement: Supplementary Figure 1 — Kaplan-Meier analysis of all-cause mortality among COPD participants based on NHHR dichotomization (A) and excluding participants with less than 3 years of follow-up (B). [file Image_1.jpeg]
